# Membrane inlet—ion mobility spectrometry with automatic spectra evaluation as online monitoring tool for the process control of microalgae cultivation

**DOI:** 10.1002/elsc.202200039

**Published:** 2023-02-28

**Authors:** Malcolm Cämmerer, Thomas Mayer, Carolin Schott, Juliane Steingroewer, Ralf Petrich, Helko Borsdorf

**Affiliations:** ^1^ Department Monitoring and Exploration Technologies UFZ Helmholtz Centre for Environmental Research Leipzig Germany; ^2^ Faculty of Mechanical Science and Engineering Institute of Natural Materials Technology, Technical University Dresden Dresden Germany; ^3^ IFU GmbH Private Institute for Analytics Frankenberg/Sa. Germany

**Keywords:** algal biotechnology, classification methods, ion mobility spectrometry, *Limnospira platensis*, process monitoring

## Abstract

The cultivation of algae either in open raceway ponds or in closed bioreactors could allow the renewable production of biomass for food, pharmaceutical, cosmetic, or chemical industries. Optimal cultivation conditions are however required to ensure that the production of these compounds is both efficient and economical. Therefore, high‐frequency analytical measurements are required to allow timely process control and to detect possible disturbances during algae growth. Such analytical methods are only available to a limited extent. Therefore, we introduced a method for monitoring algae release volatile organic compounds (VOCs) in the headspace above a bioreactor in real time. This method is based on ion mobility spectrometry (IMS) in combination with a membrane inlet (MI). The unique feature of IMS is that complete spectra are detected in real time instead of sum signals. These spectral patterns produced in the ion mobility spectrum were evaluated automatically via principal component analysis (PCA). The detected peak patterns are characteristic for the respective algae culture; allow the assignment of the individual growth phases and reflect the influence of experimental parameters. These results allow for the first time a continuous monitoring of the algae cultivation and thus an early detection of possible disturbances in the biotechnological process.

**Abbreviations**
CPC: C‐phycocyanin
LOD: limit of detection
MI: membrane inlet
IMS: ion mobility spectrometry
OD: optical density
OTR: oxygen transfer rate
UV‐Vis: Ultraviolet‐Visible spectroscopy

## 1 INTRODUCTION

The sustainable, industrial use of algae and their cell ingredients for the production of valuable compounds and active substances is a promising alternative to fossil raw materials and synthetically manufactured products [1]. Pharmaceutically relevant active ingredients and nutritionally highly effective substances such as the polyunsaturated ω‐3 fatty acids [2], natural colorants such as phycocyanin [3, 4] and chlorophyll [5], and highly potent antioxidants such as astaxanthin [6], lutein or zeaxanthin are examples for products of microalgae biotechnology. The growth in the markets for these products is driven by both regulatory changes and growing interest from consumers in natural and healthy products [7, 8, 9, 10]. In addition, the cultivation of microalgae offers benefits compared to standard agricultural production as cultivation can take place on non‐arable land and promises higher yields. The downstream processing of microalgae to yield the products is, however, often time consuming and expensive which can prevent these products from being economically viable [11]. It is therefore important to maximize the growth rate, culture cell density, and product content of the algae through careful strain selection and control of the growth conditions. Any disturbance to the culture not only reduces the product yield but can also lead to greater downstream purification costs to ensure a hazard free product. It is therefore important to closely monitor the culture to prevent losses. Consequently, such biotechnological processes require extensive process monitoring and control [12]. Most of the analytical techniques used for this purpose are *off‐line* procedures that require sample collection, sample preparation, or dilution of sample. A summary of analytical techniques that are frequently used to monitor biological processes is presented in Table 1.

**TABLE 1:** Summary of analytical techniques currently used to monitor bioreactors

	Analytical Information	Advantages	Disadvantages	Reference
Gas sensors (e.g., Respiration Activity Monitoring System RAMOS)	Partial pressure of gases (O_2_ and CO_2_)	Can calculate oxygen transfer rate (OTR) which correlates with specific growth rate	No specific information (sum signal describing metabolic rate)	[13–14, 22–23]
Electrochemical culture medium sensors	Enzyme modification leads to signal relating to concentration of a specific compound (e.g., ethanol)	Rate of production of specific product through enzyme modification (targeted analytics)	Enzyme modification affected by temperature and pH	[15, 16, 17, 18]
Absorption based optical sensors	Extinction proportional to concentration at low concentration (e.g., proteins or pigments). Attenuation of signal due to scattering can also be measured.	Cell concentration can be determined as well as qualitative information regarding photoactive compounds	Sample preparation or dilution required at higher cell concentrations.	[19–21, 24]
Scattering based optical sensors	Scattered light caused by cells	Online measurements of cell concentration possible	Disturbances by adsorption or cell agglomeration	[25, 26, 27]
Fluorescence based optical sensors	Fluorescence from specific compounds in the medium or organism	Specific product information	Sample preparation or dilution may be required at higher cell concentrations Correction factors may need to be applied	[28, 29, 30, 31, 32]

**PRACTICAL APPLICATION**

Ion mobility spectrometry (IMS) in combination with a membrane inlet (MI) was successfully introduced as tool for *online* monitoring of micro algae cultivation. The developed method bases on algae release volatile organic compounds (VOCs) in the headspace above a bioreactor in real time. The practical benefit is the possibility for predicting the optimal harvest time in systems with varying conditions such as open raceway ponds. Furthermore, early detection of possible disturbances permits the user to check the system before the product yield reduces. The ion mobility spectra are recorded every 10 min and the resulting three‐dimensional data matrix is evaluated using principal component analysis (PCA). Therefore, the developed procedure can be automated and integrated into biotechnological processes.

The oxygen transfer rate (OTR) is widely used to study the growth of microbial or plant cell cultures [13]. In phototropic algae cultivation the production of oxygen through photosynthesis is affected by the light intensity. The OTR can therefore be used to estimate the specific growth rate [14, 15, 16]. The concentration of the substrate, for example, glucose in a photomixotrophic cultivation, or the product of the cultivation, for example, ethanol, in the culture medium can also be effectively used to characterize a cultivation. This is achieved using amperometric or potentiometric sensors with enzymes immobilized on their surface [17, 18]. Specific non‐enzymatic sensors have also been developed to minimize the influence of number of factors, including temperature and pH, which can effect enzymatic sensors [19]. Whilst specific sensors such as these can provide useful data, bioreceptor (e.g., enzyme) based sensors are targeted to measure the quality of a specific compound dissolved in the culture medium [20]. Optical sensors can provide a wide range of targeted data as well as non‐targeted information relating to pigments (visible spectroscopy), biomass (light scattering), and other molecules (infrared spectroscopy) [21].

Ultraviolet/Visible spectroscopy (UV‐Vis) can be used at fairly low cost to determine the concentrations of substrates, metabolites, and products. UV‐Vis spectroscopy can also be used to estimate the concentration of C‐phycocyanin (CPC) or its purity [31]. Unfortunately, turbidity is a problem [30] meaning that as the cell concentration increases, the accuracy decreases. The fluorescence of CPC can also be used to determine its concentration but this method is also best applied to low concentrations [28, 29]. As Raman spectroscopy is more robust to sample turbidity [32], it is an alternative, if more expensive way, to characterize a wide range of substances with minimal sample pre‐treatment. However, the Raman effect is weak and, as with other optical methods, a complex matrix with multiple components leads to complex overlapping spectra [33]. The sample matrix is not only complex due to the substance but also as a result of the wide range of metabolites produced during the cultivation.

Another possible approach is the detection of algae release volatile organic compounds (VOCs) in the headspace above a bioreactor. The VOC profile can provide useful information about the metabolic processes that are taking place [33] and with a suitable analytical technique these signals can be resolved to minimize signal overlap. Due to the effect of the metabolism on the VOC profile, their study may offer the required information regarding changes to cell concentration. For example, it has been shown that the VOC profile of *Limnospira platensis (L. platensis)* [34] is influenced by the level of illumination [35]. VOCs released from the liquid have obviously a clear relationship with growth conditions. However, it is notable that few other features, like the varying physicochemical conditions of the liquid media may influence the presence and the composition of VOCs in the headspace of the photobioreactors.

The study of volatile metabolites is usually accomplished using gas chromatography coupled to mass spectrometry [36, 37]. This is not only an expensive and complex analytical technique but also a time‐ consuming step. This form of analysis can be made more economically viable by exchanging the mass spectrometer for an ion mobility spectrometer [38, 39]. Ion mobility spectrometers are typically used to detect volatile and semi‐VOCs such as explosives for security purposes and the military [40]. The instrumentation is relatively small, low power and can be used at atmospheric pressure using ambient air as the gas supply. Ion mobility measurements are based on drift velocities of ion swarms derived from sample molecules. Since the measurements are made on ions, the formation of these ions from neutral sample molecules is a first and controlling event in this method. Ionization of samples also occurs in air at ambient pressure using radioactive ionization sources, photoionization, or Corona Discharge ionization [41]. The ions are injected at a given time interval of a few microseconds via an electronic shutter into the drift region with typical lengths ranging between 5 and 15 cm. The ion packet moves as a swarm toward a detector down a voltage gradient and through a gas flow, either air or nitrogen, in a direction opposite to that of ion motion. The ion swarm has characteristic drift velocities, which provide the basis for ion separation through differences in mass and structure. Although ion mobility spectrometry (IMS) is able to provide automated, cost‐effective, and stable characterization of VOC profiles, the measurements are significantly affected by humidity in the gas sample [42, 43]. The use of gas chromatography limits the problems caused by high humidity. However, it also limits the sample volume, and therefore the limit of detection. The use of a membrane inlet is another method, which has been shown to allow the analysis of high‐humidity samples using IMS [44, 45]. Membrane inlet (MI)‐IMS is, therefore, a simple and effective method to study the VOC profile of a humid gas sample.

This study concerns the use of a custom build MI integrated into an ion mobility spectrometer to allow the automated, *in‐line* characterization of the VOC profile of a bioreactor. The response of the MI‐IMS system to changes in cell density and light intensity was then studied to analyze the effectiveness of this technique. The question was whether characteristic and reproducible signal patterns can be detected in the different growth phases, so that deviations from this pattern can be used as an indicator for possible disturbances during the cultivation due to algae associated parasites, nutrient limitation, or technical malfunction. For optimizing and testing our process analytical technology, the cyanobacterium *L. platensis* was cultivated several times with comparable results between cultivations and test locations.

The cultivation of *L. platensis* is not only of interest as a renewable source of proteins and fatty acids but also due to the biological activity of compounds produced by *L. platensis* [46], including compounds with anti‐oxidant and anti‐cancer activity [47]. Phycocyanins, linked to the proteins, are the main pigment found in *L. platensis*. Phycocyanin (approximately 15% of the protein matrix) is an antioxidant which is used as an ingredient in various products developed in the cosmetic and pharmaceutical industries. It is also used as a colorant in the food industry. Therefore, the commercial production of *L. platensis* growths throughout the world and an available analytical monitoring system would also have a high practical relevance here [12].

## 2 MATERIALS AND METHODS

### 2.1 Bioreactor

*L. platensis* PCC7345 was obtained from the Pasteur Culture Collection (PCC, Paris, France). Pre‐cultures of *L. platensis* were maintained at 30°C in 250 ml shaking flasks, which were illuminated for 18 h per day with an LED lamp (PGLE 18 RBW, Venso EcoSolutions AB, Sweden). The intensity of the lamp was adjusted to match the conditions used in the bioreactor. These pre‐cultures were rediluted with fresh modified Zarrouks culture medium twice a week so that the optical density at 750 nm (*OD*_750_) was between 0.2 and 0.6. The *OD*_750_ of the pre‐culture was measured before dilution in the main reactor to ensure a starting *OD*_750_ of 0.11 ± 0.01. The culture medium was prepared according to [48] with the following composition (L^−1^): 12.0 g NaHCO_3_ (99.5%, VWR Chemicals, Darmstadt, Germany), 0.5 g K_2_HPO_4_ (99%, Carl Roth, Karlsruhe, Germany), 2.5 g NaNO_3_ (99.9%, VWR Chemicals, Darmstadt, Germany), 1.0 g K_2_SO_4_ (99.5%, VWR Chemicals, Darmstadt, Germany), 1.0 g NaCl (99%, Carl Roth, Karlsruhe, Germany), 0.2 g MgSO_4_ˑ7H_2_O (99%, Carl Roth, Karlsruhe, Germany), 0.04 g CaCl_2_ˑ2H_2_O (99%, Carl Roth, Karlsruhe, Germany) and 100 μl Hunters trace element solution [49].

**FIGURE 1:**
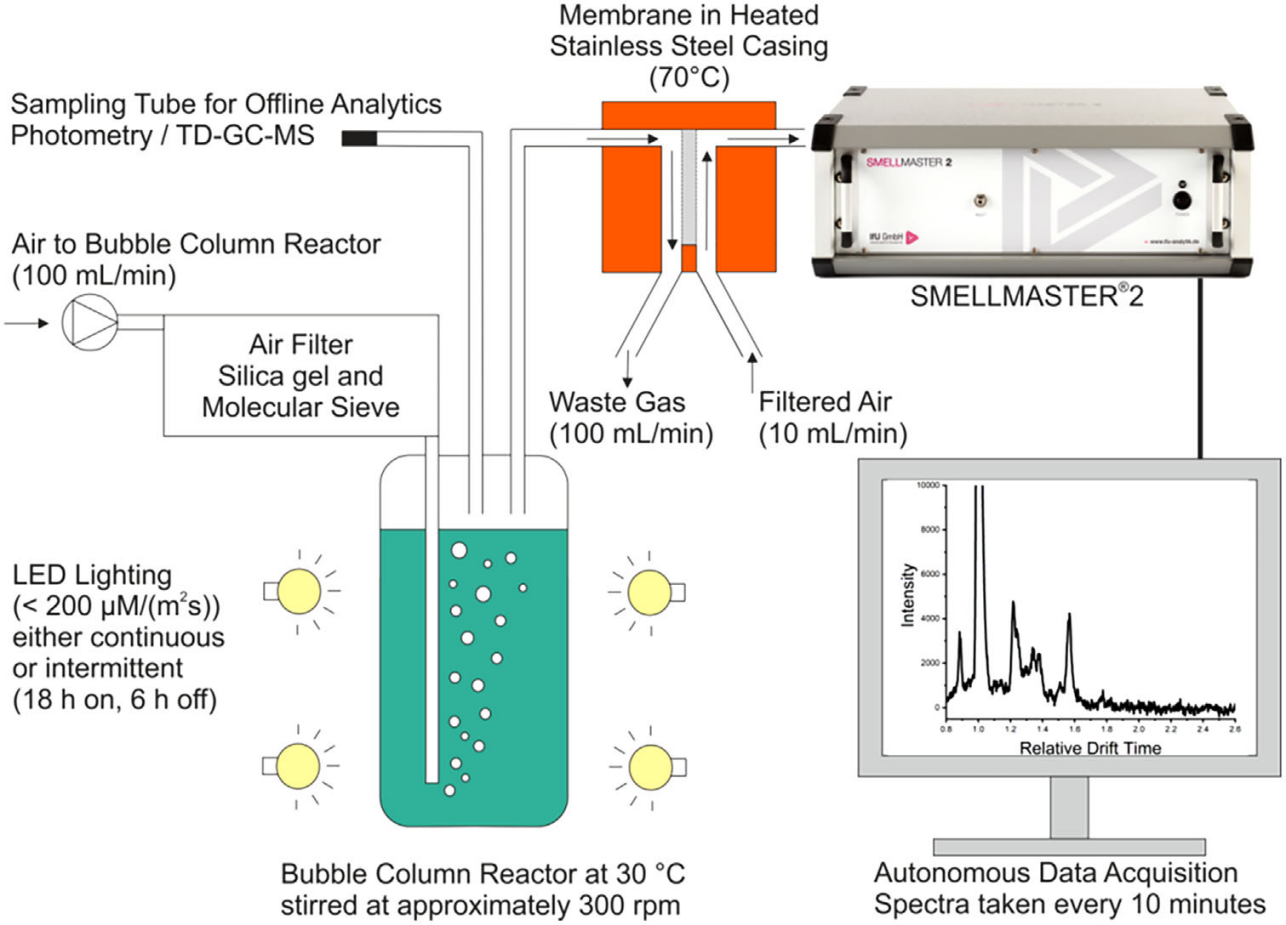
Schematic of experimental setup.

The 850 ml culture was cultured in a 1 L Duran GLS80 Laboratory Bottle (DWK Life Sciences GmbH, Wertheim, Germany). The culture medium was maintained at 30°C and stirred at a rate of 300 min^−1^. The culture was aerated with purified air at a rate of 100 ml min^−1^, which was fed directly to the MI (Figure 1).

*Chlorella vulgaris* SAG 211‐11b was obtained from the Sammlung von Algenkulturen Göttingen (SAG, Göttingen, Germany). The organisms were cultivated in Bristol Modified Medium (BMM) [50]. The four stock solutions (10 ml L^−1^ mineral salt solution, 10 ml L^−1^ nitrate solution, 10 ml L^−1^ phosphate solution, and 1 ml L^−1^ trace element solution) were mixed in a 1 L volumetric flask and filled up with distilled water for preparation of 1 L of BMM. The pH was adjusted between 6.6 and 6.8 with sodium hydroxide or hydrochloric acid. After that, the mixture was transferred into a 1 L Schott bottle and autoclaved at 121°C for 20 min.

Each cultivation was carried out at least three times at each site (Helmholtz Centre for Environmental Research in Leipzig and at the Technical University in Dresden) and the online measurements were taken at each cultivation (compare chapter 3.1).

### 2.2 Membrane inlet

The membrane cell for transition of sample molecules from the humid reactor gas stream into the inlet gas stream of ion mobility spectrometer was made from stainless steel. Both sites of the membrane module have identical channels with the following dimensions: radius 0.5 mm, depth 1 mm, width 1 mm, and length 112 mm. The exchange surface was 112.8 mm^2^. The gas streams were guided in counterflow principle through the membrane module. The gas flow from the bioreactor was pumped using a membrane pump (3003 series, Gardner Denver Thomas, Inc., Fürstenfeldbruck, Germany) with 100 ml min^−1^ and the sample gas stream into the ion mobility spectrometer was sucked by the spectrometer´s internal pump with 10 ml min^−1^. A 127 μm polydimethylsiloxane (PDMS) membrane (J‐Flex Rubber Products, Nottinghamshire, UK) was used. The membrane cell was heated to 70°C using a PWR220T‐35 Series Power Resistor (20 Ohm, 35W).

### 2.3 Ion mobility spectrometry

In drift time IMS, ions produced are separated according to the time taken to travel to the detector. Firstly, a packet of ions is generated by opening the ion gate for a short period of time, in our case 100 μs. The ions are accelerated towards the detector as a result of the electric field applied to the drift tube and reach a consistent and ion specific drift velocity. The ions are retarded by collisions with the neutral gas present in the drift tube. This means that ions are separated according to their mass, charge, and collision cross section [39, 40].

The drift time, *t_d_*, taken to reach the detector at a length, *L*, from the ion gate is characteristic for each individual ion for a particular electric field, *E*. This means that each ion can be assigned a characteristic ion mobility, *K*. Alternatively this can be standardized for the IMS operating temperature and pressure to calculate the reduced ion mobility, *K*_0_, as shown in [51].

(1) $$\;t_{d}=\frac{L}{EK}\;=\frac{L}{EK_{o}}\;\frac{T_{o}}{T}\frac{p}{p_{o}}$$

An alternative method to standardize the measurement is to normalize the drift time against the drift time of a well‐defined peak. The largest peak in an ion mobility spectrum is typically the reactant ion peak that results from the ionization of existing air components and transfer their protons to the target analytes in subsequent reactions. In positive polarity IMS this ion is a protonated water cluster, (H_2_O)_n_H^+^. As seen in [52], when the drift time is normalized against that of the reactant ion peak (arise from ionized air constituents), all instrumental factors cancel out. The relative drift time, *t_rel_*, is independent of instrumental factors which leads to a lower standard deviation in the measurement [41].

(2) $$\;t_{rel}=\frac{t_{dIon}}{t_{dRIP}}\;=\frac{K_{oRIP}}{K_{oIon}}\;$$

The measurements were performed with a Smellmaster 2 ion mobility spectrometer (IFU GmbH Privates Institut für Analytik, Frankenberg, Germany) equipped with a Tritium (^3^H) ionization source. The following operational conditions were applied for the measurements: ionization source: Tritium (50 MBq); shutter opening time: 100 μs; length of drift tube: 56 mm; drift voltage: 2470 V; polarity of ions measured: positive; drift gas: air with 340 ml min^−1^; drift tube temperature 80°C, pressure: ambient (950–1050 hPa). The spectra were taken and stored by the Smellmaster 2 every 10 min. Data analysis of these spectra was performed using custom scripts written in Python. More detailed information about the program and the corresponding scripts can be found in the Supporting Information.

### 2.4 Offline analytics with photometry

Offline photometric measurements were taken 5–6 times per day using a Dr. Lange CADAS 200 spectrometer (Dr. Bruno Lange GmbH, Berlin, Germany). A sample (<4 ml) was taken from the reactor and diluted to ensure an *OD*_750_ of less than 0.2 before the *OD*_750_ was measured in a 1 cm quartz cuvette. Fresh culture medium was added to the reactor to ensure the volume of the culture was not changed. The *OD*_750_ was measured 6 times (algae resuspended between each measurement) and a complete spectrum (400–900 nm) was taken to allow the pigment content to be estimated. The *OD*_750_ has been shown to be related to the dry weight (c_x_) of the algae using the formula shown in Equation (3) [53].

(3) $$c_{x}\;\;\left[g/L^{-1}\right]=\;0.86\cdot OD_{750}$$

## 3 RESULTS AND DISCUSSION

### 3.1 Pattern of ion mobility spectra depending on growth phase and reproducibility

**FIGURE 2:**
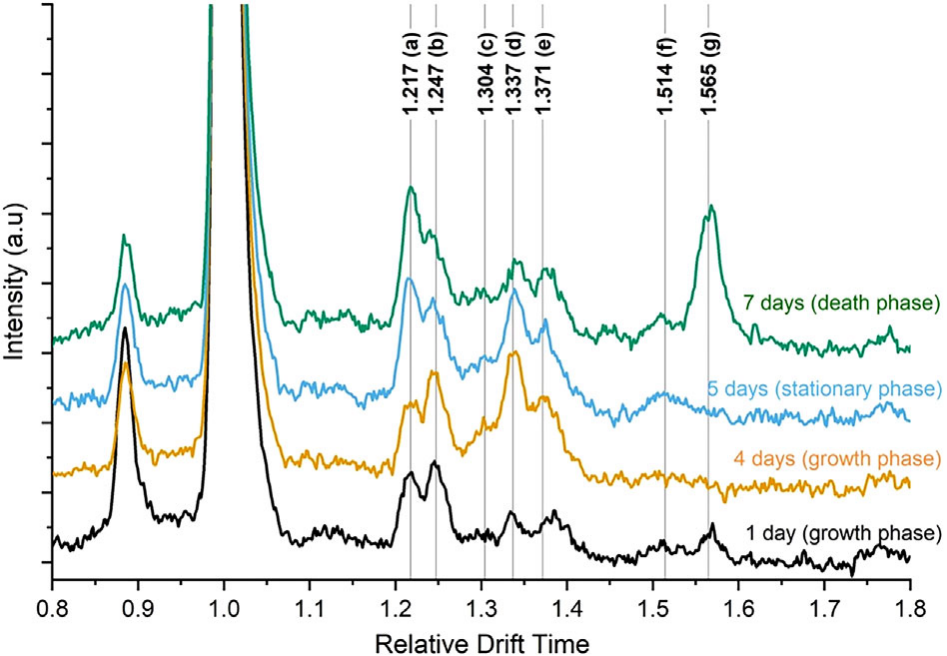
Ion mobility spectra over the course of an algae cultivation of *L. platensis*. Seven peaks identified as relevant (peaks a–g with the corresponding relative drift times).

A series of ion mobility spectra from samples, which were taken from the headspace above the bioreactor during the cultivation of *L. platensis*, is shown in Figure 2. The reactant ion peak can be seen at a relative drift time of 1 (see Equation (2)) as well as a small peak at lower relative drift times which was consistently observed, both when sampling the headspace above the algae and when flushing the ionization region of the ion mobility spectrometer with filtered air. The seven characteristic peaks marked in Figure 2 were identified by comparing the measurements taken during cultivation to the blank measurements of the cultivation medium. These peaks were observed at levels greater than the limit of detection, LOD, (3 x the background signal noise) and also seen to vary in intensity over the course of a cultivation. As these peaks were greater than the LOD, they could be easily identified using the Python function, scipy.signal.find_peaks. In general, the peaks in the middle (c, d, and e) increase in intensity during the growth phase whereas peak g increases sharply as the cell concentration in the culture reaches a maximum. It is clearly shown that the ion mobility spectra and thus the composition of the VOCs change over the course of the cultivation. Each phase of cultivation shows significant pattern in ion mobility spectra. Before the experiment started, the ion mobility spectra of pure culture medium were taken. This ensured that no contamination was present as contamination of the culture medium, for example as a result of ineffective cleaning of the reactor between experiments, led to a signal in the ion mobility spectrum which differed from the typical blank spectrum.

The stability of the MI‐IMS system was demonstrated by comparing the ion mobility spectra taken from cultivations of *L. platensis* performed at the Helmholtz Centre for Environmental Research in Leipzig and at the Technical University in Dresden. Both research groups used an identical equipment including bioreactor, sampling position, MI, and coupling to the ion mobility spectrometer. The peak positions of peaks a–g were accurately reproduced when sampling the headspace above identical *L. platensis* cultures using two Smellmaster 2 ion mobility spectrometers. These results can be found in the Supporting Information (Figure S1). The pattern of peaks also varied in a similar manner with peaks c, d, and e rising during the growth period and peak g rising sharply at the end of the cultivation. The similarity in the changes in peak intensity over the course of an 8‐day cultivation can be observed. This shows that, independent of the location, user and instrument used, IMS can reproducibly track the changes in the VOC profile of a microalgae cultivation.

**FIGURE 3:**
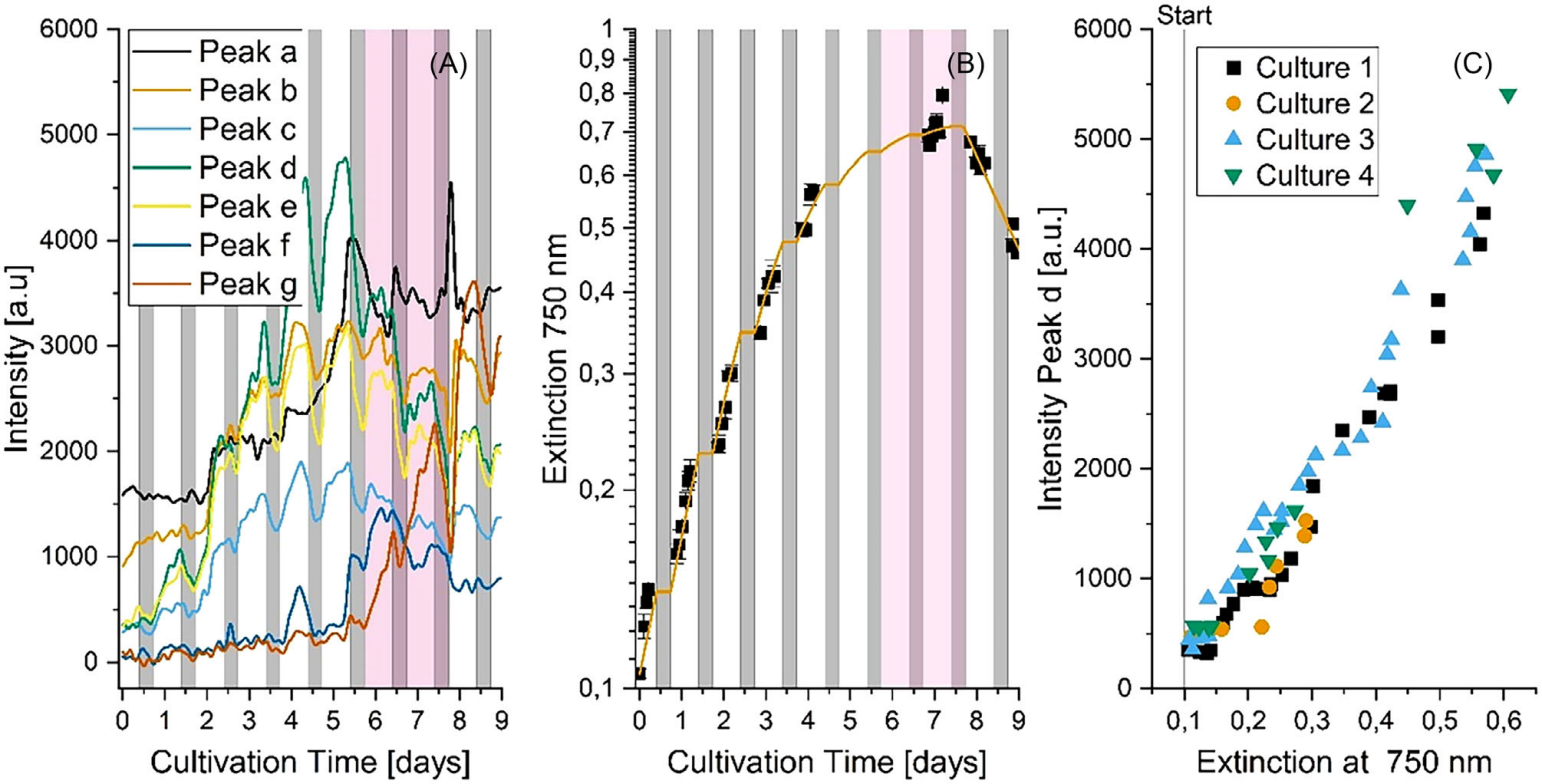
The maximum peak intensities of seven peaks (A) and changes in the extinction measured at 750 nm (B) over the course of an *L. platensis* cultivation. Gray bars show the times when the illuminations was turned off (8 h per day). The pink bar shows the stationary growth phase. Correlation of the intensity of peak d to *OD*_750_ (C).

The intensities of the seven relevant peaks over the course of cultivation can be seen in Figure 3A and compared to the cell concentration determined from the *OD*_750_ (Figure 3B). Comparing peak d to the *OD*_750_ measurements, the maximum intensity is reached just before the growth curve levels out and enters the stationary phase (marked pink). This trend is mirrored by peaks c and e to a lesser extent. These three peaks all show a strong light dependence. Peaks c, d, and e rise when the culture is illuminated and growing (days 0–5 in white marked areas) and then drop when the illumination is turned off (gray marked areas). Finally, a new peak, peak g, is observed as the cultivation reaches a maximum cell concentration at around day 6. This peak continues to increase as the cell concentration starts to decrease.

These trends in peak intensities were consistently observed across different cultivations performed under identical conditions. The cultures all started with an *OD*_750_ of 0.11 ± 0.01 and an increase in cell concentration was observed until day 5. The maximum cell concentration was then reached between day 5 and 6 before a decrease in cell concentration was observed. The maximum intensity of peak d was consistently measured on day 5 or 6 and was normally observed just before the maximum cell concentration was achieved. This indicates that peak d is a marker for the growth rate and when the maximum cell concentration has been reached. The intensity of peak g was seen to rise in all cultures from the onset of the stationary growth phase into the cell death phase. The presence of peak g above a threshold value can, therefore, be used in process control to bring an end to the cultivation as the culture has now started to die.

Changes in peak intensity can be used to approximate the cell concentration in the growth phase. The intensity of peak d rises in the first days of cultivation. In Figure 3C it can be seen that the intensity of peak d also correlates with the *OD*_750_ (*R* ^2^ > 0.95). This correlation shows both that peak d is an important peak in the peak pattern associated with growth, but also that the MI‐IMS based system works effectively at low cell concentrations. This is in contrast to other online sensors such as scattered light sensors. These sensors are used to measure the growth of a culture; however, they normally require higher cell concentrations to show a clear correlation between the sensor signal and the cell concentration. The backscattered signal is often disturbed by absorption or cell agglomeration [27].

A student's parametric t‐test was performed to check whether the intensity changes of peaks a–g were significant changes over the growth phases. The results of this can be found in Table S1 of the Supporting Information. For this purpose, the experimental data of 4 independent cultivations were evaluated. We compared the changes in intensity of separate peaks a–g over the cultivation with that at the begin of the experiment (start time). As can be seen from Table S1 (Supporting Information), the differences of peak intensities can be considered as statistically significant.

**FIGURE 4:**
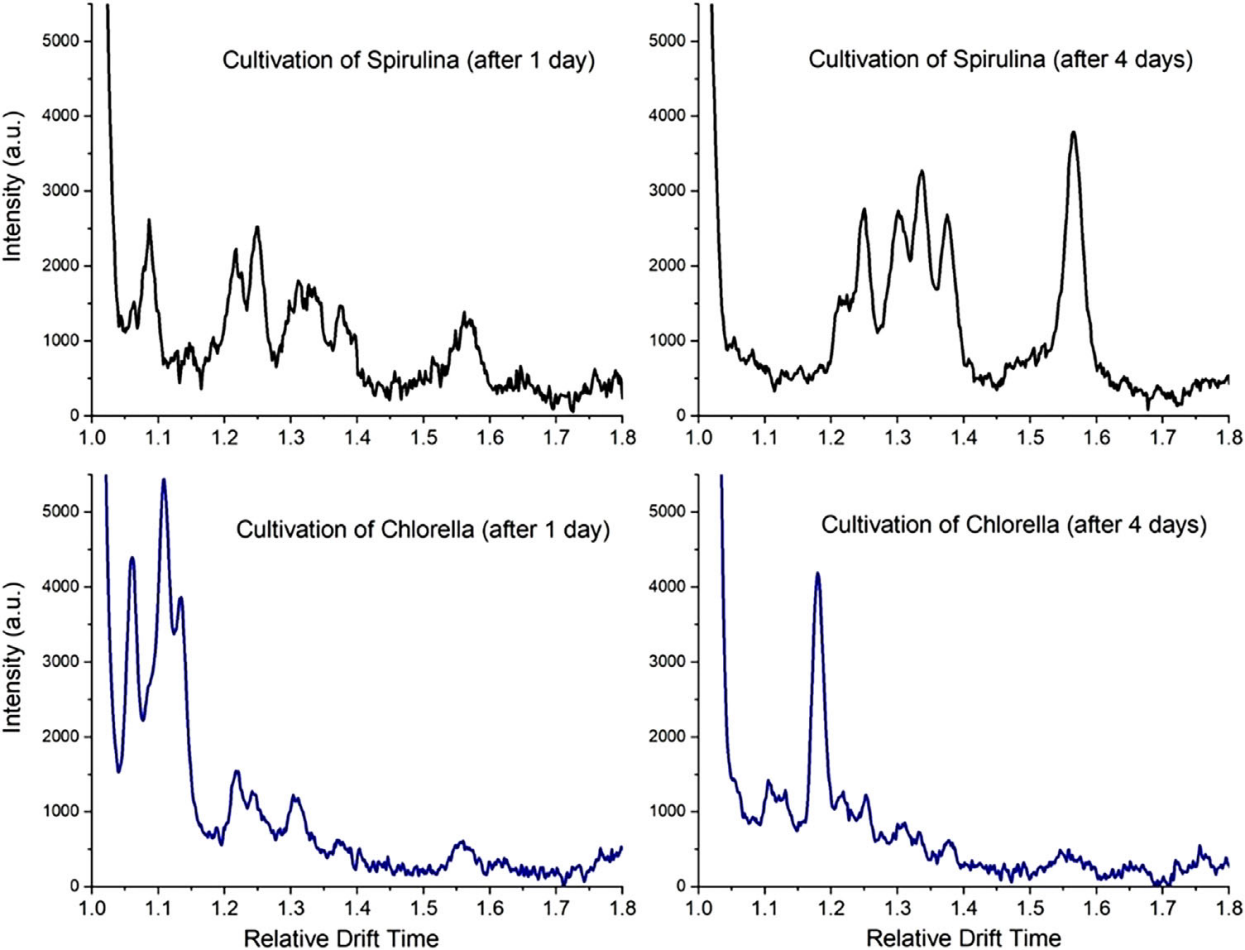
Ion mobility spectra taken on the first and fourth day of cultivation of *L. platensis* (Spirulina) (black) and *C. vulgaris* (Chlorella) (blue).

Whilst microalgae share similar metabolic pathways, a simple proof of concept to test the suitability of IMS for measuring the VOCs produced by microalgae was to sample the headspace above two different microalgae cultivations. As seen in Figure 4, the ion mobility spectra of *L. platensis* (Spirulina) and *C. vulgaris* (Chlorella) differ significantly from each other. Peaks a–g can clearly be observed in the two ion mobility spectra of Spirulina between relative drift times of 1.2 and 1.6. By contrast, the major peaks in the two ion mobility spectra of Chlorella occur before 1.2. This shows that the ion mobility spectrometer has sufficient resolving power to differentiate the VOC profiles of two different microalgae. Although this work focuses on using IMS as on *online* monitoring tool for the process control to maximize production, these results show the potential for using IMS to detect contaminations, such as grazers, in a cultivation.

As a result of these investigations it could be shown that (1) different microalgae cultivations lead to fundamentally different spectra. (2) The intensity ratios of the peaks within the ion mobility spectra significantly vary over the course of an algae cultivation. (3) The cultivation of the same species of microalgae yields identical and reproducible ion mobility spectra and comparable intensity courses during cultivation.

From these results, some general practical conclusions can be drawn for the application of IMS with respect to information about the bioprocess: (1) When selected peaks reach their maximum intensity, the maximum growth rate is reached. (2) The peak intensities (in particular at low cell concentrations) correlate with cell concentration. (3) A drop in all growth‐related peaks without new peaks normally reflects the day/night cycle. (4) The appearance of new peaks in the ion mobility spectrum indicates change in cell functions, for example, nutrient limitation or lysis.

### 3.2 Principal component analysis

While individual peaks can be correlated effectively with changes in the cell concentration, it has already been shown that certain peaks, for example, peaks c–e, correlate strongly with each other. In addition, there are over 1000 data points within the ion mobility spectrum corresponding to relative drift times between 0.7 and 3. A peak is therefore made up of multiple data points which, under ideal conditions, lead to a gaussian peak shape in the spectrum. This leads to two different strategies to process the data to maximize the number of data points used in the analysis and minimize the influence of signal noise. The data can be modeled using a combination of multiple gaussian peaks with fixed relative drift times. Alternatively, principal component analysis (PCA) can be used to identify the peaks which correlate with each other and build principal components (PC). In both cases, all data points after the reactant ion peak are included in the data set. This means that, for each spectrum, the intensity measured at over 1000 relative drift time points is evaluated as opposed to the maximum intensities of, in this case, seven peaks. In the gaussian peak model, the data matrix is compared to a model built using peaks with a specific center point and width. This allows the noise in the measurement of the intensity to be reduced and, in this case, seven more reliable data points to be generated for each spectrum in the time series. Using PCA, the dimensionality of the data set is reduced from more than 1000 x number of spectra in the time series (**** x ****) to a data set of, in this case, **** x 4. Reference data are then used to link either the 4 PC Vectors or the seven peaks from the gaussian model to a recommendation for the process control.

**FIGURE 5:**
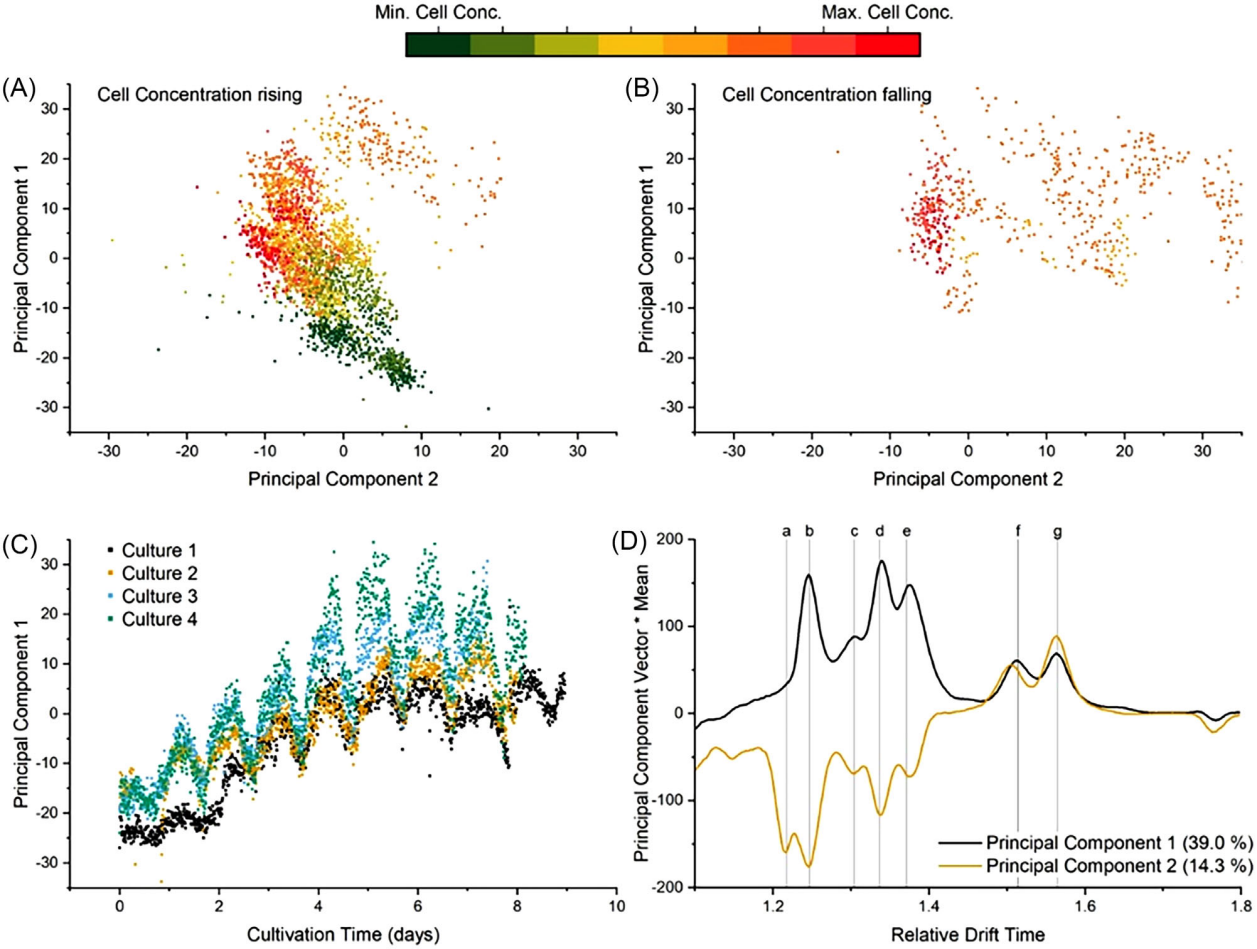
Results of PCA showing the scores for PC 1 and 2 for IMS spectra taken in cultures 1 to 4 when the cell concentration was rising (A) and when the cell concentration was falling (B). The color‐coding is normalized over the complete data set. The variation in PC 1 scores with respect to time for the individual cultures is also shown (C). The contribution of peaks a–g to the PC scores is graphically represented (D) by multiplying the PC Vector with the mean intensity for each data point.

A custom Python code was written using the sklearn package (see Supporting Information). The standard scaler was used before passing the data to the PCA for analysis using with four PC. PCA was used in this work to analyze the spectra from six cultivations. Four different cultures (1–4) were performed under identical conditions whilst two additional cultures (5 and 6) experienced low‐level illumination over the first day of the cultivation and thereafter high‐level illumination (60% → 200% intensity compared to cultures 1–4). A growth phase was observed in all six cultures over multiple days followed by a short stationary phase and period of cell death (as seen in Figure 5A). The initial expectation was therefore that the first PC would represent the growth phase due to the large change in cell concentration and therefore VOC production over multiple IMS spectra. Furthermore, the second PC was expected to represent the stationary / cell death phase due to the presence of a new peak, peak g.

As seen in Figure 5A and B, both of these expectations were realized. PC 1 scores increase from −30 to 25 as the cell concentration increases. During this time, only a minor variation in PC 2 scores can be observed. As the cell concentration starts to drop, PC 2 scores increase from −10 to 30. Furthermore, variation in PC 1 scores due to the light source being turned on and off can also be observed in Figure 5C. Although the change in PC1 for Culture 1 in Figure 5C was different to the other cultures with respect to time, it can be seen in Figure 5A that the data points for low cell concentration (colored green) are reasonably tightly grouped. If we assume that, the production of VOC is linked to the cell concentration and the stage of growth, then it is reasonable to allow a small variation in the PC1 level with respect to time. The variation in PC 1 scores is caused by the influence of peaks c, d, and e, as shown in Figure 5D. High peak intensities for peaks c, d, and e lead to a high PC 1 score. It was previously shown that these peaks are associated with growth and their intensities decrease in the absence of light. As PC 2 is associated with negative growth, it is logical that peaks c–e are correlate negatively with PC 2. In addition, peak g, which was previously linked to cell death, correlates positively with PC 2.

These results can be used as a basis for process control. New spectra taken can be characterized using the PC transformation obtained from the training data set. The process control system can then give recommendations or automatically trigger control events. (1) If PC 1 rises and PC 2 remains constant, the culture is growing and the cell concentration is increasing. Therefore, no changes in the system are necessary. (2) If PC 1 reaches maximum and PC 2 remains constant, the culture achieved maximum cell concentration and the practical recommendation is: Harvest or add more culture medium. (3) If PC 2 increases, the cell concentration is decreasing. The recommendation from process control system would be: Harvest, add more culture medium or check for contamination. (4) If PC 2 is strongly negative, there is an unknown system status and the user is prompted to check the system.

### 3.3 Influence of light on cultivation

Illumination is essential for the phototrophic growth of the algae cultures. In all the cultures presented, *L. platensis* uses the carbonate ions in the culture medium as a carbon source to produce sugars from photosynthesis which then allow growth. As already shown, the absence of illumination leads to a reduction in peak intensities in the IMS spectra and also a reduction in PC 1. It should not however be assumed that higher levels of illumination lead to increased growth rates. Above a species‐specific maximum level of illumination, the photosystems in *Arthrospira sp*. are saturated and the rate of growth does not increase. Furthermore, under intense illumination, concentrations of CPC decrease [54, 55, 56], which harms product yields of this high‐value antioxidant. In the case of laboratory experiments, the level of illumination is carefully controlled. Industrial scale microalgae cultivations, however, rely on the sun to provide illumination. Depending on the construction of the reactor this could lead to the level of illumination being either too high or too low. As low illumination reduces the growth rate, a system, which shades the reactor when the illumination is too high, provides the optimum growth conditions without the additional cost of artificial illumination. This shading system would require process control, which could be automatically controlled using the level of illumination and the current cell concentration for a bioreactor with known dimensions. All these parameters are necessary, as the microalgae shade themselves. This means that even at constant illumination levels, microalgae experience different levels of illumination depending on the cell concentration. Alternatively, changes in the VOC profile as a result of over illumination can be used for process control.

**FIGURE 6:**
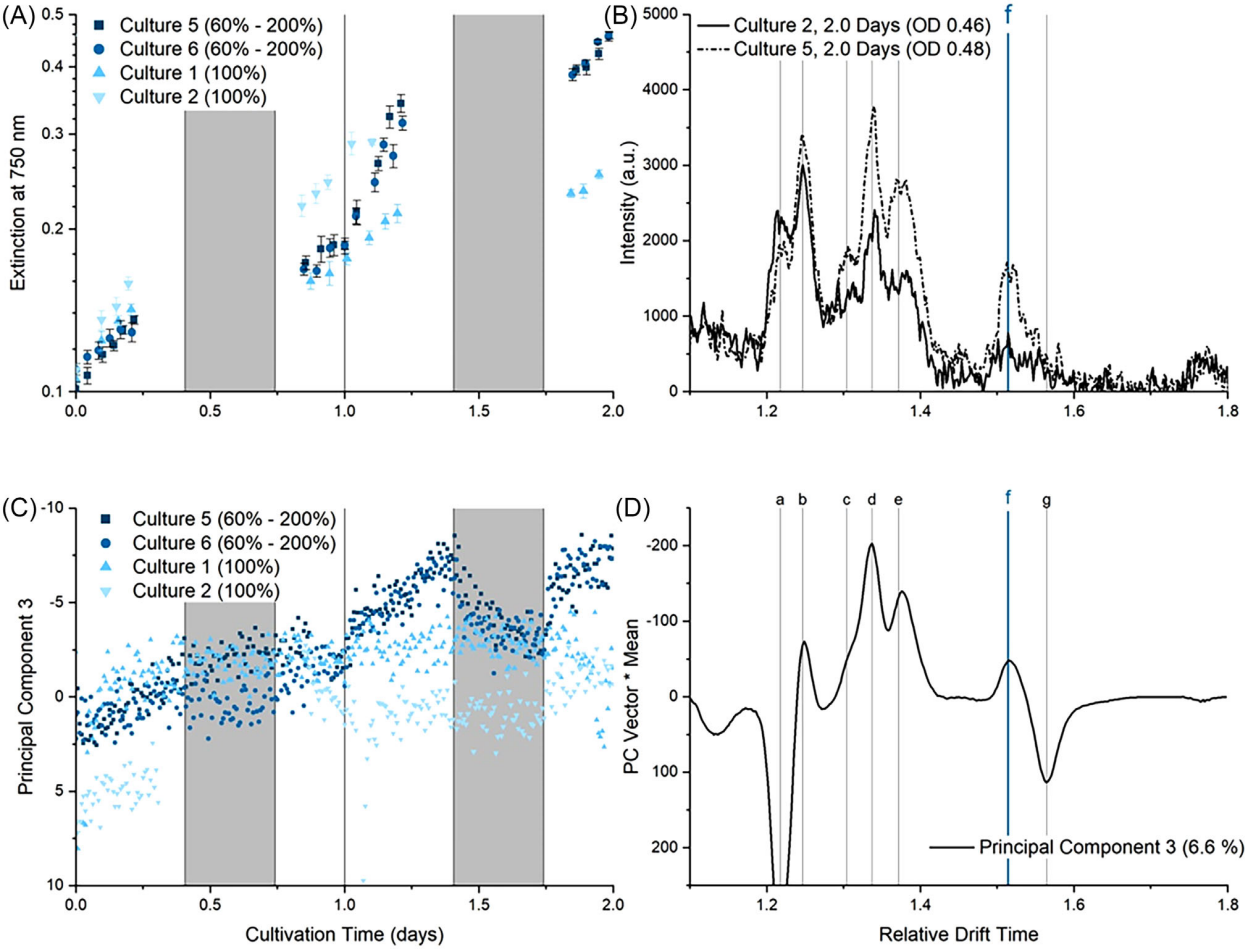
Effect of varying the level of illumination on the extinction at 750 nm (A), the IMS spectra measured after 2 days (B) and the PC 3 scores. The contribution of peaks a–g to the PC 3 scores is graphically represented (D) by multiplying the PC 3 Vector with the mean intensity for each data point. In cultures 5 and 6 the initial illumination was 60% (relative to culture 1–4). The illumination was increased after 1 day to 200%.

Cultivations 5 and 6 were therefore performed under low and high levels of illumination and the changes in the IMS spectra and PC scores were observed. As seen in Figure 6A, increasing the level of illumination leads to an increase in the growth rate of cultures 5 and 6. As a result, peaks c–e are significantly more intense when comparing the IMS spectra in Figure 6B. Peak f is also observed under high illumination leading to a new pattern of peaks. This new pattern of peaks, peaks c, d, e, and f correlated, is seen in PC 3 and shown in Figure 6D. The change in PC 3 scores can therefore be used to track periods of extreme illumination as PC 3 changes rapidly as a result of the increased illumination. This almost instantaneous response to high illumination is shown in Figure 6C and leads to a new process control recommendation: Shade culture when PC 3 < −5.

The presence of peak f, and obviously a change to the metabolic processes under high illumination, could result from the degradation of CPC for use as a nitrogen source. Further investigation is however required to determine if the addition of a further nitrogen source to the culture medium prevents peak f from forming and allows high levels of growth without CPC degradation under high illumination.

## 4 CONCLUDING REMARKS

IMS equipped with a MI is a promising technology to allow reliable and accurate process monitoring and control of microalgae cultivations. The VOCs can be automatically sampled when the IMS is coupled in‐line and data acquisition occurs every 10 min with zero “hands‐on” time required from an operator. This high frequency of measurement allows process control to occur in a timely manner. The automated, cost‐effective and stable VOC spectra acquired from MI‐IMS can then be used to characterize the condition of the cultivation and provide recommendations for the process control. PCA was performed on a training data set of 6 cultures and the first three PCs could be used to determine: if the culture was growing; if the maximum cell concentration had been reached; if the cell concentration was decreasing and if the level of illumination was too high. This application of a single sensor system to provide multiple process control signals sets MI‐IMS apart from the analytical techniques listed in Table 1. When applied to commercial production, process control using MI‐IMS can be used to increase yields, aid detection of contaminations and allow optimization of growth rates.

## CONFLICT OF INTEREST STATEMENT

The authors declare no competing interests.

## Supporting information

Supplementary Figure 1. Comparison of IMS‐spectra from two cultivations of *L. platensis* performed on identical equipment at different locations
Supplementary Table 1: Results of statistical analysis

## Data Availability

The data that support the findings of this study are available from the corresponding author upon reasonable request.
