# Supplementary material for: Membrane inlet—ion mobility spectrometry with automatic spectra evaluation as online monitoring tool for the process control of microalgae cultivation
Source: Eng Life Sci. 2023 Feb 28;23(4):e2200039. doi: 10.1002/elsc.202200039 (PMC10071569; doi:10.1002/elsc.202200039)
Supplement: Supplementary file 1 — Supplementary Figure 1. Comparison of IMS‐spectra from two cultivations of L. platensis performed on identical equipment at different locations Supplementary Table 1: Results of statistical analysis [file ELSC-23-e2200039-s001.docx]

**Supporting Information**

**Membrane inlet - ion mobility spectrometry with automatic spectra evaluation as online monitoring tool for the process control of microalgae cultivation**

Malcolm Cämmerer^1^, Thomas Mayer^1^, Carolin Schott^2^, Juliane Steingroewer^2^, Ralf Petrich^3^, Helko Borsdorf^1^

^1^ UFZ Helmholtz Centre for Environmental Research, Department Monitoring and Exploration Technologies, Permoserstraße 15, D-04318 Leipzig, Germany

^2^ Technical University Dresden, Faculty of Mechanical Science and Engineering, Institute of Natural Materials Technology, Bergstraße 120, D-01069 Dresden, Germany

^3^ IFU GmbH Private Institute for Analytics, An der Autobahn 7, 09669 Frankenberg/Sa., Germany


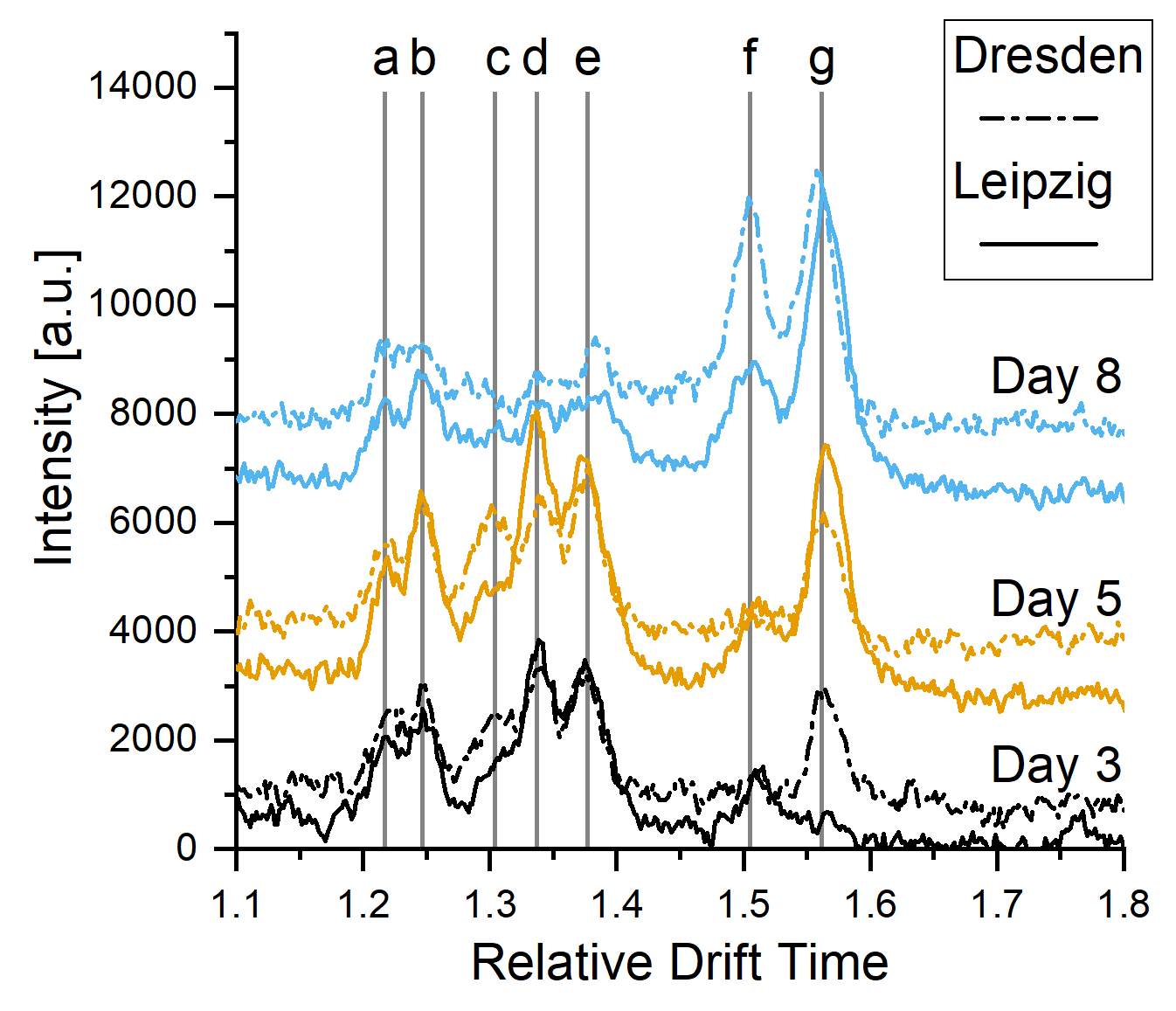


**Supplementary Figure 1.** Comparison of IMS-spectra from two cultivations of *L. platensis* performed on identical equipment at different locations. The pattern of peaks vary in a similar manner with peaks c, d and e rising during the growth period and peak g rising sharply at the end of the cultivation.

**Supplementary Table 1:** Results of statistical analysis

**Phyton-Scripts**

**Script 1:**

1) Open a folder

2) Open each individual file

a) Finds the reactant ion peak using find_peaks

b) Creates a gaussian model of the reactant ion peak and fits this (lmfit)

c) Uses the modelled peak time to transform the drift time to relative drift time

d) Maps the data point onto predefined relative drift times so that all data files have same relative drift time scale

e) Add these data point to the data matrix (pandas)

3) Uses the file created time from the first_culture_file to create a relative timestamp for each spectrum

4) Saves two excel files

a) All Spectra in a matrix of Relative Drift time and cultivation time

b) The filenames with their respective cultivation times

'''

Gauss parameters a,b and c are peak height, position and sigma

FWHM = 2*np.sqrt(2*np.ln(2))* sigma

Use lmfit minimise to model all peaks simultaneously

see https://millenia.cars.aps.anl.gov/software/python/lmfit/examples/example_fit_multi_datasets.html

'''

#siems et al. model factors

kBoltzmann=1.38065e-23

elemantarladung=1.602e-19

#tail factors from Peak form.xlsx

f_a = 0.26

f_b = 0.061

f_c = 1.58

w_min = 1.2124e-4

factor = 7.0982e-5

#User Entry - set folder and first_kultur_file

ordner = 'Y:\\Gruppen\\met\\PPM\\Labordaten 2022\\MoProAlge\\220221 1liter Kutlur LED foil'#'Y:\\Gruppen\\met\\PPM\\Labordaten 2021\\MoProAlge\\Blasensäulenreaktor LED\\0811 1liter Kultur LED'#'Y:\\Gruppen\\met\\PPM\\Labordaten 2022\\MoProAlge\\Dresden Ergebnisse\\KG_Medium_08.02.22'#

first_Kultur_file = '2102 alge 5v45%-0001.p.json'#'0811 Alge-0001.p.json'#'KG8.2-0001.p.json'#'ASP06_IMS-0001.p.json'#'ASP04_IMS-0001.p.json'#'2009 d1 Algen-0001.p.json'#

gif_title = 'Relative Driftzeit 210222'

files = os.listdir(ordner)

files.sort()

check_for_excel = False

Data = pd.DataFrame(columns = ['Filenames', 'Pump_1'])

Peaks = None

red_mobs = None

Spectren = None

settings_df = None

setting_items ={"AccelerationVoltage","AirPressure","TubePressure","TubeTemperature","PumpCircFlow",'SamplingInterval_us'}

old_spec = np.ones(21)

rep_counter = 0

rep_check = False

time_stamp = 0

light_on= True

zero_time = None

t_start = time.perf_counter()

#function, objection, and final model for gauss peak with tail

def fit_gauss_plus_tail(x,a,b, w, fac):

c = np.sqrt(np.square(b)*fac + w)

fit = a*(np.exp(-(x-b)**2/(2*c**2))) + f_a*a*(np.exp(-(x-(b+f_b))**2/(2*(f_c*c)**2)))

#second gauss peak models tail

return fit

def gauss_tail_dataset(params, i, x):

#Calculate Gaussian lineshape from parameters for data set.#

amp = params['amp_%i' % (i+1)]

cen = params['cen_%i' % (i+1)]

w = params['w_min']

fac = params['factor']

return fit_gauss_plus_tail(x, amp, cen, w, fac)

def objective_gaus_tail(params, x, data, LOQ, no_params):

#compare the model to the data

resid = data

for i in range(no_params):

model_i = gauss_tail_dataset(params, i, x)

if np.any(np.isnan(model_i)):

print('Model number %d contains NaN' % (i+1))

#remove NaN by converting to zeros

#Nans tend to exist before 1ms (see test in fit_gauss_plus_tail)

model_i[np.isnan(model_i)] = 0

resid = resid - model_i

#print(np.sum(np.square(resid)))

return resid

def final_model_gaus_tail(params, x, no_params):

#create final model

model = np.zeros_like(x)

for i in range(no_params):

model = model + gauss_tail_dataset(params, i, x)

return model

def zero_data_json(data, data_name):

offset = data.loc[0:300, data_name].mean()

noise = data.loc[0:300, data_name].std()

return data.sub([offset,0]), noise

def data_analysis(data, data_name, factor, gate, RIP_time, rel_time_set):

#zero baseline and get step size and noise level

data, noise = zero_data_json(data, data_name) #peaks come after 3.5ms

#data.plot(x='time', y=data_name)

min_height = noise*3 # 3x standard deviation

data['residual'] = data.loc[:,data_name]

peak_df = None

all_peak_df = None

loops = 0

#set min and max for RIP find

min_x = 4.5

max_x = 5.1

time_index = pd.Index(data['time'].values)

min_loc = time_index.get_loc(min_x, method = 'nearest')

max_loc = time_index.get_loc(max_x, method = 'nearest')

#find peaks based on max min FWHM and min height level

while np.amax(data.loc[min_loc:max_loc,'residual'].max()) > min_height:

peaks = find_peaks(data.loc[min_loc:max_loc,'residual'].to_numpy().flatten(), height=min_height, prominence=noise*3)

peaks_pos = peaks[0]+min_loc

if type(peak_df) is type(None):

peak_df = data.iloc[peaks_pos,:].copy()

peak_df['c'] = np.sqrt(np.square(peak_df.loc[:,'time'].values)*factor + w_min)

elif len(peaks[0]) == 0:

break

else:

peak_df = data.iloc[peaks_pos,:].copy()

peak_df['c'] = np.sqrt(np.square(peak_df.loc[:,'time'].values)*factor + w_min)

for i in peaks_pos:

data.loc[min_loc:max_loc,'residual'] = data.loc[min_loc:max_loc,'residual'].values-fit_gauss_plus_tail(data.loc[min_loc:max_loc,'time'].values, peak_df.at[i,'residual'], peak_df.at[i,'time'], factor, gate)

if type(all_peak_df) is type(None):

all_peak_df = peak_df.copy()

else:

all_peak_df = all_peak_df.merge(peak_df, how='outer')

loops+=1

if loops ==10:

break#set peak heights for peaks between 3.5 and 12.5 (absolute time)

if len(all_peak_df.index)<1:

print('no peaks found')

return None

else:

all_peak_df.reset_index(drop=True, inplace=True)

#fit peak find model peaks with tail using lmfit

fit_params = Parameters()

for iy in range(len(all_peak_df.index)):

amp = all_peak_df.at[iy,'residual']

cen = all_peak_df.at[iy,'time']

sig = np.sqrt(np.square(all_peak_df.at[iy,'time'])*factor + w_min)

#fit params based on measured peak

if amp > min_height:

fit_params.add('amp_%i' % (iy+1), value=amp*0.8, min=0, max=amp*2)

elif amp < 0:

fit_params.add('amp_%i' % (iy+1), value=0, vary=False)

else:

fit_params.add('amp_%i' % (iy+1), value=amp, vary=False)

fit_params.add('cen_%i' % (iy+1), value=cen, min=cen-3*sig, max=cen+3*sig)

fit_params.add('w_min', value=9.58e-5, vary=False)# min=1e-5, max=1e-3)

fit_params.add('factor', value=6.05e-5, vary=False)# min=1e-5, max=1e-3)

#print(fit_params.pretty_print())

#reduce dataset between min and max peaks

x = data.loc[:,'time'][data.loc[:,'time']>min_x].to_numpy()

y = data.loc[:,data_name][data.loc[:,'time']>min_x].to_numpy()

y = y[np.where(x < max_x)]

x = x[np.where(x < max_x)]

#reduce dataset (only include data less than 55000)

x = x[np.where(y < 55000)]

y = y[np.where(y < 55000)]

try:

out2 = minimize(objective_gaus_tail, fit_params, args=(x, y, min_height, len(all_peak_df.index)), method='leastsq', max_nfev=100000)

except ValueError as e:

print(str(e))

data.plot.line(x=data_name+'(ms)',y=list((data_name)))

plt.xlim([min_x-1,max_x])

plt.show()

return None

if out2.success:

print("Peak Find Model\nFitting finished after %d iterations" % (out2.nfev))

#print(out2.params.pretty_print())

coeffs = pd.DataFrame.from_dict(out2.params.valuesdict(), orient='index', columns=['a'])

coeffs.drop(index=['w_min','factor'], inplace=True)

col_names = ['amp', 'cen']

coeffs['col_names'] = matlib.repmat(col_names,1,len(all_peak_df.index)).flatten()

coeffs['index_ids'] = matlib.repmat(np.arange(1,len(all_peak_df.index)+1),2,1).T.flatten()

coeffs = coeffs.pivot(index='index_ids', columns='col_names')

coeffs.columns = coeffs.columns.droplevel()

#find RIP greater than 4.7 and less than 5.2

try:

#print(coeffs.loc[:,:][coeffs.loc[:,'cen'].gt(4.7) & coeffs.loc[:,'cen'].lt(5.2)])

rip_pos = coeffs.at[coeffs.loc[:,'amp'][coeffs.loc[:,'cen'].gt(4.5) & coeffs.loc[:,'cen'].lt(5.1)].idxmax(),'cen']

print('RIP at %.4f ms, IfU RIP at %.4f ms' % tuple((rip_pos, float(RIP_time))))

except:

print('no RIP found, IfU RIP at %.4f ms' % float(RIP_time))

rip_pos = float(RIP_time)

return None

data['relative time'] = data.loc[:,'time']/rip_pos

coeffs['relative time'] = coeffs.loc[:,'cen']/rip_pos

#create model using peaks

data['abs_model'] = final_model_gaus_tail(out2.params, data.loc[:,'time'].to_numpy(), len(all_peak_df.index))

data.plot.line(x='time',y=list((data_name, 'abs_model')))

plt.xlim([min_x-1,12])#max_x])

plt.vlines(rip_pos,0, data['abs_model'].max())

plt.show()

if type(rel_time_set) is type(None):

# create standard relative time array

standard_RIP = 5 #position of standard RIP in ms

min_loc = time_index.get_loc(4, method = 'nearest')

max_loc = time_index.get_loc(13, method = 'nearest')

rel_time_array = data.loc[min_loc:max_loc,'time'].values / standard_RIP

else:

rel_time_array = rel_time_set

#interpolate rel_time data

f2 = interp1d(data['relative time'].values, data.loc[:,data_name], kind='cubic', fill_value='extrapolate')

rel_time_int = f2(rel_time_array)

df_dict ={'rel_time':rel_time_array, 'residual':rel_time_int}

rel_time_df = pd.DataFrame.from_dict(df_dict).set_index('rel_time')

return rel_time_df

else:

print('did not converge')

return None

def open_data_json(filename):

global rep_counter, old_spec

filepath =(ordner,filename)

filepath = "\\".join(filepath)

extension = os.path.splitext(filename)[-1].lower()

name = os.path.splitext(filename)[0][:-2]

try:

extension =='.json'

f = open(filepath)

val = json.load(f)

#print('file open')

try:

file_timestamp = time.strptime(val['DeviceSettings']["LocalTime"], "%Y-%m-%dT%H:%M:%S+02:00")

except:

file_timestamp = time.strptime(val['DeviceSettings']["LocalTime"], "%Y-%m-%dT%H:%M:%S+01:00")

file_timestamp_s = time.mktime(file_timestamp)

#print('got time')

light_on = file_timestamp[3]>5 and file_timestamp[3]<21

# get device settings

device_settings = {k: v for k, v in val['DeviceSettings'].items() if k in setting_items}

#print('device settings')

# check voltage

if device_settings['AccelerationVoltage'] < 2000:

print('Voltage too low')

raise Exception

device_settings['Pump1Flow'] = val['Positive']['Pump1Flow']

device_settings['timestamp'] = (file_timestamp_s-zero_time)/(60*60*24) # timestamp in hours

print('filename: %s, time in days: %.3f' % tuple((name,device_settings['timestamp'])))

device_settings['lamp'] = light_on

device_settings = pd.DataFrame.from_dict(device_settings,orient='index',columns=[name])

#spectrum and test to see if it's different to the last

spectrum = pd.DataFrame(val['Positive']['DataAsU16'],columns =[name])

#print(spectrum.head())

if np.all(spectrum.loc[0:20,name].values == old_spec):

rep_counter += 1

print('Spectrum repeat')

raise Exception

else:

rep_counter = 0

old_spec = spectrum.loc[0:20,name].values

# get IfU RIP

RIP_time = val['Positive']['RipDriftzeit_ms']

#create time signal

try:

x = np.arange(val['Positive']['X0'],val['Positive']['X1']+0.0001,device_settings.at['SamplingInterval_us', name]/1000)

spectrum['time'] = x

rel_time = False

except:

x = np.linspace(val['Positive']['X0'],val['Positive']['X1'],2048)

spectrum['rel_time'] = x

rel_time = True

return name, spectrum, device_settings, RIP_time, rel_time

except:

print('Filename: %s skipped' % filename)

return None, None, None, None, None

def save_data_func(coeffs, mobs, name):

data_name='.'.join((name[0:-4],'xlsx'))

data = {'a': coeffs[:,0],'b': coeffs[:,1],'c': coeffs[:,2], 'Ko': mobs}

df = pd.DataFrame(data)

df.to_excel(data_name, index=None, header=True)

print('Data saved: ',data_name)

def data_set_anal(files):

global zero_time

data_df = pd.DataFrame()

filenames = {}

#open first kultur file to get zerotime

filepath =(ordner,first_Kultur_file)

filepath = "\\".join(filepath)

try:

#time_stamp = os.path.getmtime(filepath)

f = open(filepath)

val = json.load(f)

try:

file_timestamp = time.strptime(val['DeviceSettings']["LocalTime"], "%Y-%m-%dT%H:%M:%S+02:00")

except:

file_timestamp = time.strptime(val['DeviceSettings']["LocalTime"], "%Y-%m-%dT%H:%M:%S+01:00")

file_timestamp_s = time.mktime(file_timestamp)

zero_time = file_timestamp_s

print('zero time: %.1f' % zero_time)

except:

'first data file wrong/not present'

#check if data is partially analysed

if (check_for_excel and 'rel_time_data.xlsx' in files):

filename = '\\'.join((ordner,'rel_time_data.xlsx'))

data_df = pd.read_excel(filename)

data_df.set_index('rel_time', inplace=True)

print('relative time file opened')

filename = '\\'.join((ordner,'times and filenames.xlsx'))

filenames_df = pd.read_excel(filename)

filenames = filenames_df.set_index(filenames_df.columns[0]).T.to_dict(orient='records')[0]

t_max = filenames_df['times'].max()

print('time file opened')

else:

print('no excel files')

t_max=np.NINF

#go through files

for filename in files:

data_name, data, meta_df, RIP_time, x_rel_time = open_data_json(filename)

rel_times = None

try:

if t_max >= float(meta_df.at['timestamp',os.path.splitext(filename)[0][:-2]]):

print('file skipped as data already present')

data = None

rel_times = None

except:

continue

# extract indiviual curves and analyse

if type(data) != type(None):

if len(data_df.columns)>0:

rel_time_set = data_df.index.values

else:

rel_time_set = None

if x_rel_time:

data['time'] = data.loc[:,'rel_time'].mul(RIP_time)

data.drop(columns=['rel_time'], inplace=True)

rel_times = data_analysis(data, data_name, factor, w_min, RIP_time, rel_time_set)

else:

rel_times = data_analysis(data, data_name, factor, w_min, RIP_time, rel_time_set)

if type(rel_times) is not type(None):

rel_times.columns = [meta_df.at['timestamp',os.path.splitext(filename)[0][:-2]]]

if len(data_df.columns)>0:

data_df = data_df.join(rel_times,how='left')

else:

data_df = rel_times.copy()

filenames[data_name] = meta_df.at['timestamp',os.path.splitext(filename)[0][:-2]]

else:

print('No Data for %s' % filename)

filenames_df = pd.DataFrame.from_dict(filenames, orient='index')

filenames_df.columns = ['times']

return data_df, filenames_df

### USER INPUT ####

save_summary = True

### USER INPUT ENDS ###

data_df, filenames = data_set_anal(files)

if save_summary:

filename = '\\'.join((ordner,'rel_time_data.xlsx'))

data_df.to_excel(filename, header=True)

print('relative time file saved')

filename = '\\'.join((ordner,'times and filenames.xlsx'))

filenames.to_excel(filename, header=True)

print('time file saved')

else:

print('file(s) not saved')

**Script 2:**

1) Open the excel files created with json_spektren_to_excel.py

2) Add the spectra to a pandas dataframe

3) Performs PCA on the combined data set

4) Saves an excel file with the data from PCA

data_dict = {}

stand_devs = {}

pca_df = None

shorten_dataset = True

for key in files_dict.keys():

print('Opening {} now'.format(key))

filename = '\\'.join((files_dict[key],'rel_time_data.xlsx'))

data_df=pd.read_excel(filename)

data_dict[key] = data_df.copy().set_index('rel_time')

print("length of {} index: {}".format(key,len(data_df.index)))

stand_devs[key] = data_df.std(axis=1).values

suffix = ''.join(('_',key))

if pca_df is None:

pca_df = data_df.copy().set_index('rel_time').add_suffix(suffix)

else:

pca_df = pca_df.join(data_df.set_index('rel_time').add_suffix(suffix))

stand_devs = pd.DataFrame.from_dict(stand_devs)

stand_devs.set_index(data_df.set_index('rel_time').index, inplace=True)

#sns.lineplot(data=stand_devs.iloc[180:610], dashes=False) # only use data between 1.15 and 1.8 (there are only 6 levels of dashes)

sns.lineplot(data=stand_devs.iloc[275:675], dashes=False) # for dresden results

plt.show()

names_array = np.array(pca_df.columns.values, dtype=str)

names_array = np.char.split(names_array, sep ='_')

names_df = pd.DataFrame(np.stack(names_array), columns=['time', 'runID'])

names_df['time'] = names_df['time'].astype("float32")

#Run PCA

pca = PCA(n_components=4)

#shorten dataset?

if shorten_dataset:

pca_df = pca_df.iloc[180:610] # for leipzig results

print("length of dataset is shortened to: {}".format(len(pca_df.index)))

x = StandardScaler().fit_transform(pca_df.T.values)

pc_amps = pca.fit_transform(x)

pc_amps_df = pd.DataFrame(data = pc_amps, columns = ['PC1', 'PC2', 'PC3', 'PC4'])

pc_amps_df['time']=names_df['time']

pc_amps_df['runID']=names_df['runID']

sns.scatterplot(data=pc_amps_df, x='PC1', y='PC2', hue='time', style='runID')

plt.show()

#create MultiIndex

index = pd.MultiIndex.from_arrays([pc_amps_df['runID'].values, pc_amps_df.index.values])

pc_amps_df.set_index(index, inplace=True)

#print individual graphs:

for name in pc_amps_df['runID'].unique():

graph_data = pc_amps_df.loc[name]

no_kg = graph_data.loc[:,'time'] > 0

sns.scatterplot(data=graph_data.loc[no_kg], x='PC1', y='PC2', hue='time', style='runID')

plt.xlim([-40,40])

plt.ylim([-40,40])

plt.show()

total_sd = pca_df.std(axis=1)

total_mean = pca_df.mean(axis=1)

components_df = pd.DataFrame(data=pca.components_.T, columns = ['PC1', 'PC2', 'PC3', 'PC4'], index=pca_df.index)

components_sd_df = pd.DataFrame(data=pca.components_.T, columns = ['PC1', 'PC2', 'PC3', 'PC4'], index=pca_df.index).multiply(total_sd, axis=0)

components_mean_df = pd.DataFrame(data=pca.components_.T, columns = ['PC1', 'PC2', 'PC3', 'PC4'], index=pca_df.index).multiply(total_mean, axis=0)

for pc in ['PC1', 'PC2', 'PC3', 'PC4']:

sns.relplot(data=pc_amps_df, x='time', y=pc, hue='runID', marker=".", kind="scatter")

#plt.ylim([-40,40])

#plt.xlim([0,4])

plt.show()

sns.lineplot(data=components_df.multiply(total_sd, axis=0).reset_index(), x='rel_time', y=pc)

plt.xlabel('Relative Drift Time')

plt.show()

#save PCA components

ordner = 'Y:\\Gruppen\\met\\PPM\\Labordaten 2021\\MoProAlge\\Dresden Ergebnisse'

filename = '\\'.join((ordner,"dresden PCA short.xlsx"))

with pd.ExcelWriter(filename) as writer:

pc_amps_df.set_index('time').to_excel(writer, sheet_name='All Data', header=True)

for name in pc_amps_df['runID'].unique():

pc_amps_df.loc[name].set_index('time').to_excel(writer, sheet_name=name, header=True)

components_df.to_excel(writer, sheet_name='Components', header=True)

components_sd_df.to_excel(writer, sheet_name='Comps x SD', header=True)

components_mean_df.to_excel(writer, sheet_name='Comps x Mean', header=True)

print("Explained variance (%)\nPC1: {:0.1f}%\nPC2: {:0.1f}%\nPC3: {:0.1f}%\nPC3: {:0.1f}%".format(*pca.explained_variance_ratio_*100))
